# The Fundamental Biological Factors Underlying the Incidence of Tuberculosis.—II

**Published:** 1922-03

**Authors:** R. J. Ewart

**Affiliations:** (Medical Officer of Health and Tuberculosis Officer, Barking Town, E.)


### THE FUNDAMENTAL BIOLOGICAL FACTORS UNDERLYING THE INCIDENCE OF TUBERCULOSIS.—II.

By R. J. EWART, M.D., D.Sc., F.R.C.S. (Medical Officer of Health and Tuberculosis Officer, Barking Town, E.)

THE conclusion can be safely drawn, that a healthy man is not likely to be prejudiced or his chance of dying of phthisis enhanced by marriage to a woman who is already consumptive. As a corollary to this it may be stated that infection must occur before adult life in all cases. Evidence has already been given which seems to show that the process of infection is complete by the fourteenth year.

It is not unreasonable, therefore, to surmise that the factor which decides whether immunity can be established with or without discomfort, and if with discomfort, whether death supervenes or not, is the constitution or diathesis that is inherited from the parents. It has been conclusively shown that, with equal opportunity of infection, a son of a tuberculous father is much more likely to die of consumption than the sister of a tuberculous brother. The association is of the same order in each case as that of any physical inherited character.

The correlation between father or mother and child for phthisis is $r=0{\cdot}5$, the same as for eye colour, &c., and for members of the same sibship (that is brother and sister), it is about 0·4 in each case. These refer to completed life histories, but the same is found amongst children. The following data bearing on this point was collected in the area, it refers to the association of lethal tuberculosis in the family, and existance of recognisable pulmonary tuberculosis in a child under 7 and 14 years, the child being in school attendance.

BARKING SCHOOL CHILDREN.

| GROUP 5 TO 7 YEARS OLD. | No recognisable disease. | With recognisable disease. | Total. |
|---|---|---|---|
| No family history | 2,048 | 32 | 2,086 |
| Father or mother having died or likely to die of phthisis | 257 | 25 (6) | 282 |
| Totals ... | 2,305 | 57 | 2,362 |

$r=0{\cdot}42\pm0{\cdot}01.$

As a matter of chance we should have expected to find six infected children of tainted stock; in actual fact we have 25, that is four times too many. There is, of course, some error in these figures calculated to increase the value of the association.

| GROUP 13 YEARS OLD. | | Child. | | |
|---|---|---|---|---|
| | | With disease. | Without disease. | Total. |
| Parent | With disease | 29 | 127 | 156 |
| | Without disease | 20 | 1,302 | 1,322 |
| Totals ... | | 49 | 1,429 | 1,478 |

$r=0{\cdot}59\pm0{\cdot}02.$ (?)

That is, in seven years the association between parent and offspring increases from 0·42 to 0·59. This gives some statistical evidence that lends support to the tuberculin tests previously quoted, with respect to the progress of reaction according to age.

Taking now brother and sister, where the infection factor must be as strong as the foregoing, we have :—

| | | Boys. | | |
|---|---|---|---|---|
| | | Not tuberculous. | Tuberculous. | Total. |
| Girls | Not tuberculous | 1,836 | 93 | 1,929 |
| | Tuberculous | 87 | 12 (5) | 99 |
| Totals ... | | 1,923 | 105 | 2,028 |

$r=0{\cdot}20\pm0{\cdot}02.$ (?)

Thus, out of 2,028 families that contain one boy and girl, 192 had at least one boy or girl with recognisable signs of tuberculosis, and in 12 one boy and girl, the number expected as a matter of chance was five, that is, about half the relationship between parent and child.

These figures are of some importance if compared amongst themselves. The variation in the criterion of what a School Medical Officer considers to be recognisable tuberculosis in a child, is so great that comparison with other series or from an absolute standpoint is hopeless. It is seen that in children as age advances, the inherited factor becomes more marked, and probably by the fourteenth year is completely established, and that, with equal opportunity of infection, a son of a consumptive father is more likely to suffer than a brother of a similarly affected sister. Hence the importance of the diathesis, that is, wherever infection is universally present, diathesis must be the factor in deciding the nature and time of reaction.

### TREATMENT.

A word as to the result of treatment, as seen locally, may not be without interest.

Much discontent has arisen lately owing to the fact that tuberculosis is still with us. It is to be regretted that the enthusiasts of ten to fifteen years ago claimed too much for their proposals, so that now there is a risk of an organisation being prejudiced which has something to be said for it. Anyone who has had charge of a sanatorium or any knowledge of its work must be impressed with the improvement in health that occurs as a result of the more open life, though much the same, of course, can be said for a seaside holiday.

There is, however, not sufficient data locally to assess the value of such treatment. The test is, what is the mean length of the after-life-time of people so treated ? As far as can be judged at present, it seems as if about 50 per cent. are dead within four years, a figure which is much the same for all notifications. It is admitted that advanced cases receive little benefit, and, in so far as there is a large error of diagnosis in early cases, a statistical difficulty is introduced that makes it almost impossible to measure with any degree of accuracy the beneficial effects of the treatment recommended.

In the five years, January, 1915, to December, 1920, 124 deaths of patients attending the dispensary were noted; in that period 246 deaths occurred from pulmonary tuberculosis (including other forms the figure was 327). That is, approximately one-third of the total population suffering from tuberculosis comes under the jurisdiction of the dispensary.

A comparison between those who have gone to sanatoria and those who have not received such treatment completely fails, because in the latter class mus t be included a number who are notified as suffering from phthisis after the death has occurred. Beyond this, acute phthisis running a rapid course cannot be dealt with in that manner, owing to the nursing required, though an attempt was made to admit these cases into the local isolation hospital. Confining oneself to those notified and still living, after four years in the former class 70 per cent. were returned as fit for some occupation and about 60 per cent. of the latter. The difference for the reason given cannot be fairly assigned to different methods of treatment. It is significant, however, that from a statistical point of view it might be assumed that the populations were similar.

Perhaps it may be of use to make a few deductions from the foregoing relative to their bearing on administrative action :—

(1) All, with a few exceptions, are infected by about the fourteenth year. (Tuberculin tests.)

(2) After that date the infection factor can be ignored. (Conjugal phthisis.)

(3) The main factor that decides the ultimate fate is the disposition or diathesis inherited from the parents.

(4) In a small number the modern methods of open-air life may enable a permanent immunity to be established.

(5) There is no statistical evidence to suggest that the permanent segregation of all notified cases of pulmonary tuberculosis would lead to a material reduction in the numbers dying from this disease. This is based on the fact that discomfort and infection are far from being synonymous. As an actual fact the infected and apparently healthy are many times more numerous and live for a much longer period of time than those showing unmistakable signs of disease; the former comprise the whole population, the latter only a section of it. The activities (and hence the power of distributing the bacillus) of the former are unrestricted, the movements of the latter are necessarily considerably curtailed by their condition.

(6) As a deduction it would seem that beyond the ordinary amenities that civilised life requires, little can be done. It certainly seems unjust to collect the dying in a leper-house for a crime of which they have not been proved guilty.

(7) Specific treatment, probably through the injection into the systems of all children in early life of some immunising substance on lines similar to vaccination, seems to be the main point of the future, but the time is not yet.

It is of interest to note that experience in the vaccination of the population against cholera, enteric fever, smallpox and plague, suggest that it is not necessary completely to immunise each individual or all individuals to prevent spread. Thus, the ideal may not be so difficult as was at one time thought.

It will be noticed that nothing has been said relative to the possibility that the cow, through the various food products obtained from that animal, may be the source of infection in man. To put the matter briefly, it may be stated that the type of parasite found in each is distinct, though each variety may be found in either. Further, it is a fundamental principle of Isolation Hospitals and the handling of all infectious ailments, that if an individual is attacked by any variety of a particular organism, he is immune to any further infections of all varieties of that organism. This is not strictly true, but is sufficiently so for administrative purposes; hence the segregation of all varieties of *measles, scarlet fever, diphtheria, &c., in separate wards.* Now if the two commonest varieties of Tubercle Bacilli (there are many others) compete for the occupation of a particular human being, that derived from a human source is, in the majority of cases, implanted on the growing child before the bovine variety, and the latter is probably prejudiced on that account alone. As a consequence the number of cases which seem to be due to a bovine variety are not so numerous as that derived from human sources. In so far as all mixed milk and much of the meat can be regarded as infected, and the cooking in many instances is not sufficient to kill the organism, the dosage must be very massive, yet it produces a relatively small effect. The bovine variety is probably not an important factor, because the available field has been previously occupied by the human variety.

It would seem that the significance of tubercle bacilli in milk may be overrated, and so long as human tuberculosis occupies the position it does it must assume a subsidiary position, but should the human variety be controlled without alteration of immunity, then the bovine variety would probably take its place and cause just as much discomfort to us. It is for this reason that food as a probable source of infection has not been considered.

### CIVIC EDUCATION LEAGUE.

Last year the Civic Education League organised a very interesting Easter visit to Belgium for the purpose of civic study. This year a similar visit to Holland is being arranged. Anyone interested in civic studies may join the party. Early application should be made to Miss Margaret Tatton, Secretary, Civic Education League, Leplay House, 65 Belgrave Road, S.W.1.

It is proposed to leave London on Thursday, April 13, and to return on Thursday, April 27. Visits will be paid to Rotterdam, The Hague, Leyden, Haarlem, Amsterdam, Utrecht and possibly Dordrecht and Middleburg. There will also be opportunities of seeing rural life in several parts of the country.

Members of the party will have special facilities for first-hand contact with the work and personnel of social and economic institutions. Education and municipal, commercial and political life will be studied in this manner. Special attention will be given to horticulture and to the art galleries and museums. Architecture, including present-day housing developments, will be studied. Mr. Alexander Farquharson, M.A., will be in charge of the educational arrangements throughout.

